# The cellular bases of mobility from the Study of Muscle, Mobility and Aging (SOMMA)

**DOI:** 10.1111/acel.14129

**Published:** 2024-03-01

**Authors:** Steven R. Cummings, Paul M. Coen, Luigi Ferrucci

**Affiliations:** ^1^ San Francisco Coordinating Center California Pacific Research Institute San Francisco California USA; ^2^ Department of Epidemiology and Biostatistics University of California San Francisco California USA; ^3^ Translational Research Institute, AdventHealth Orlando Florida USA; ^4^ Intramural Research Program of the National Institute on Aging, NIA, NIH Baltimore Maryland USA

**Keywords:** autophagy, denervation, mobility, oxidative stress, physical fitness, skeletal muscle, strength

## Abstract

Findings from the Study of Muscle, Mobility and Aging (SOMMA) in this issue of Aging Cell show that several biological pathways in skeletal muscle cells play an important role in determining mobility in older adults. These are based on assays in skeletal muscle biopsies obtained from participants, aged 70 years and older in SOMMA tested for association with assessments related to mobility, including muscle mass, strength, power, cardiopulmonary fitness, and 400 m walking speed. The papers show that, using mass spectrometry, oxidative modifications of proteins essential to myocellular function are associated with poorer mobility. Using RNA‐seq to quantify gene expression, lower levels of expression of antioxidant enzymes located in mitochondria, autophagy, patterns of expression of genes involved in autophagy, and higher levels of RNA transcripts that increase with denervation were associated with poorer performance on tests of mobility. These results extend previous research from the Baltimore Longitudinal Study of Aging and recent studies from SOMMA showing the importance of mitochondrial energetics in mobility. Together, these findings are painting a picture of how fundamental cellular processes influence the loss of mobility with aging. They may also be a window on aging in other cells, tissues, and systems. The data collected in SOMMA are publicly available and SOMMA welcomes collaborations with scientists who are interested in research about human aging.

## Graphical abstract

This issue of Aging Cell includes results from the Study of Muscle, Mobility and Aging (SOMMA) about cellular processes involved in mobility in older people. Adding to previous studies from SOMMA and from the Baltimore Longitudinal Study of Aging (BLSA) about the the important role of mitochondrial energetics, these papers find associations of oxidative modification of myocelluar proteins, antioxidant enzymes, autophagy and denervation with lower strength, fitness, and muscle mass. These represent the value of cellular epidemiology in the study of human aging.

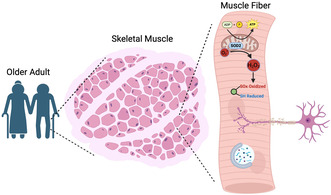

**Abbreviations**
ATP: Adenosine 5′‐triphosphate
ATPmax: Maximum capacity to generate ATP derived from ^31^P‐magnetic resonance spectroscopy of quadriceps muscle
BLSA: Baltimore Longitudinal Study of Aging
CT: Computed tomography
maxOXPHOS: Maximum consumption of oxygen in synthesis of ATP
MR: Magnetic resonance
RNA‐seq: RNA sequencing
ROS: Reactive oxygen species
SOD2: Superoxide dismutase 2

Several studies have examined the declines in muscle mass and strength to understand the underlying mechanism of mobility loss with aging. These studies have generally used measures of mobility assessed through interviews and questionaries and in some cases, performance based assessments of physical function and related them to circulating biomarkers to draw inferences about phenotypes involved. This area of research is making substantial progress by applying new paradigms and technique of cellular epidemiology that draws specific hypotheses about aging from the understanding biological processes in cells and uses assays of the cellular processes to between understand causal pathways that lead to disability with aging. Cellular epidemiology is rooted on techniques that were originally developed in cell biology and in animal models but apply them to large populations of sufficient size and power to relate fundamental cellular processes to physical and cognitive performance, and clinical outcomes. Important, this new branch of science requires strong collaboration between basic scientists with expertise in cell biology and epidemiologists who have the expertise to apply measurements from laboratories to adequately powered and well‐phenotyped human studies (Kuller et al., 2013). Cellular epidemiology proposes has the potential to identify biological mechanisms of aging that are common to cells with the proposition that discoveries about human aging at the cellular level may generalize across cells in many tissues. This approach may identify new targets for interventions aimed at preventing of slowing down physical and cognitive decline with aging.

This issue of Aging Cell carries four papers about the cellular basis of mobility from the Study of Muscle, Mobility and Aging (SOMMA), which can be considered quintessential examples of cellular epidemiology. SOMMA is a unique study of mobility and aging because it includes measurements of fundamental cellular processes of aging performed in human muscle biopsies of the vastus lateralis in a large cohort 879 participants, aged 70 years and older (Cummings et al., 2023). The study focused on a core set of measurements of muscle and mobility‐skeletal muscle mass and muscle volume, muscle strength and power, fitness by peak VO_2_ from treadmill testing, and 400 m walking speed but also collected other phenotypic measures that are relevant to aging.

Previous papers from SOMMA focused on mitochondria. They used respirometry from mitochondria in biopsies of the vastus lateralis to characterize mitochondrial energetics, primarily the maximum consumption of oxygen in synthesis of ATP for energy (maxOXPHOS). These studies showed that higher maxOXPHOS was strongly associated with better muscle power and cardiorespiratory fitness (Mau et al., 2023) and with less time to complete a 400 m walk speed (Cummings et al., 2024). These are consistent with discoveries from a smaller forerunner study for SOMMA (Coen et al., 2013). Additionally, lower levels of maxOXPHOS were associated with fatigability (Qiao et al., 2023) and frailty (Mau et al., 2024) but not the risk of falling (Kramer et al., 2024).

These results from SOMMA also confirm pioneering research from the Baltimore Longitudinal Study of Aging (BLSA). Using noninvasive P31 MRS to measure the capacity of mitochondria to generate ATP from ADP, BLSA showed that greater mitochondrial capacity to generate ATP (ATPmax) in muscle was associated with walking speed, especially when measured in tasks that require acceleration or endurance, and also predicted future changes of walking speed over time (Choi et al., 2016; Tian et al., 2022). Subsequently, in a mediation analysis, it was shown that the association between mitochondrial oxidative capacity and walking speed was significantly explained by muscle function (Zane et al., 2017). These data are consistent with proteomic studies in muscle biopsies showing that mitochondrial proteins are the class of muscle proteins most underrepresented in older age, with evidence that maintaining a high level of physical activity may offset this trend. More recently, data from the BLSA also demonstrated that mitochondrial oxidative capacity assessed in skeletal muscle predict future development of mild cognitive impairment and dementia and are associated with biomarkers of inflammation and neuroinflammation (Tian et al., 2022; Zampino et al., 2020). These findings suggest that mitochondrial function plays a central role in mediating the effect of aging in muscle, and likely also in other tissue. SOMMA collected data that already allows to expand on this hypothesis of the centrality of mitochondrial to aging and as more analyses will be done on its extremely rich database, may further shed light on it.

The papers in this issue of Aging Cell test hypotheses that other cellular properties, in addition to mitochondrial energetics, are associated with assessments of muscle and mobility (Figure 1). Two test hypotheses that originate from Harman's, (1956) proposed Free Radical Theory of Aging which posits that aging results from production of oxygen radicals (reactive oxygen species–ROS) by mitochondria. ROS that are not sufficiently neutralized by antioxidant enzymes damage essential elements of cells generally (Harman, 1956, 1972). It had been proposed that this process may promote aging in muscle (Fougere et al., 2018). Day et al. (2024) uses mass spectrometry‐based redox proteomics in a sample of SOMMA participants to find that oxidative modifications of proteins essential to myocellular function are associated with decreased performance in several measurements of mobility. In a complementary analysis, Tranah et al. (2023) used RNAseq of SOMMA muscle tissue, to show that lower levels of expression of antioxidant enzymes located in mitochondria, such as SOD2, reduce mitochondrial energetics—maxOXPHOS—as would be expected as ROS damaged mitochondria themselves. Lower levels of transcripts for antioxidant enzymes were associated reduced measurements of mobility. These are the first human studies to provide support for predictions from the Free Radical Theory for muscle and mobility (Rodney et al., 2016).

**FIGURE 1:**
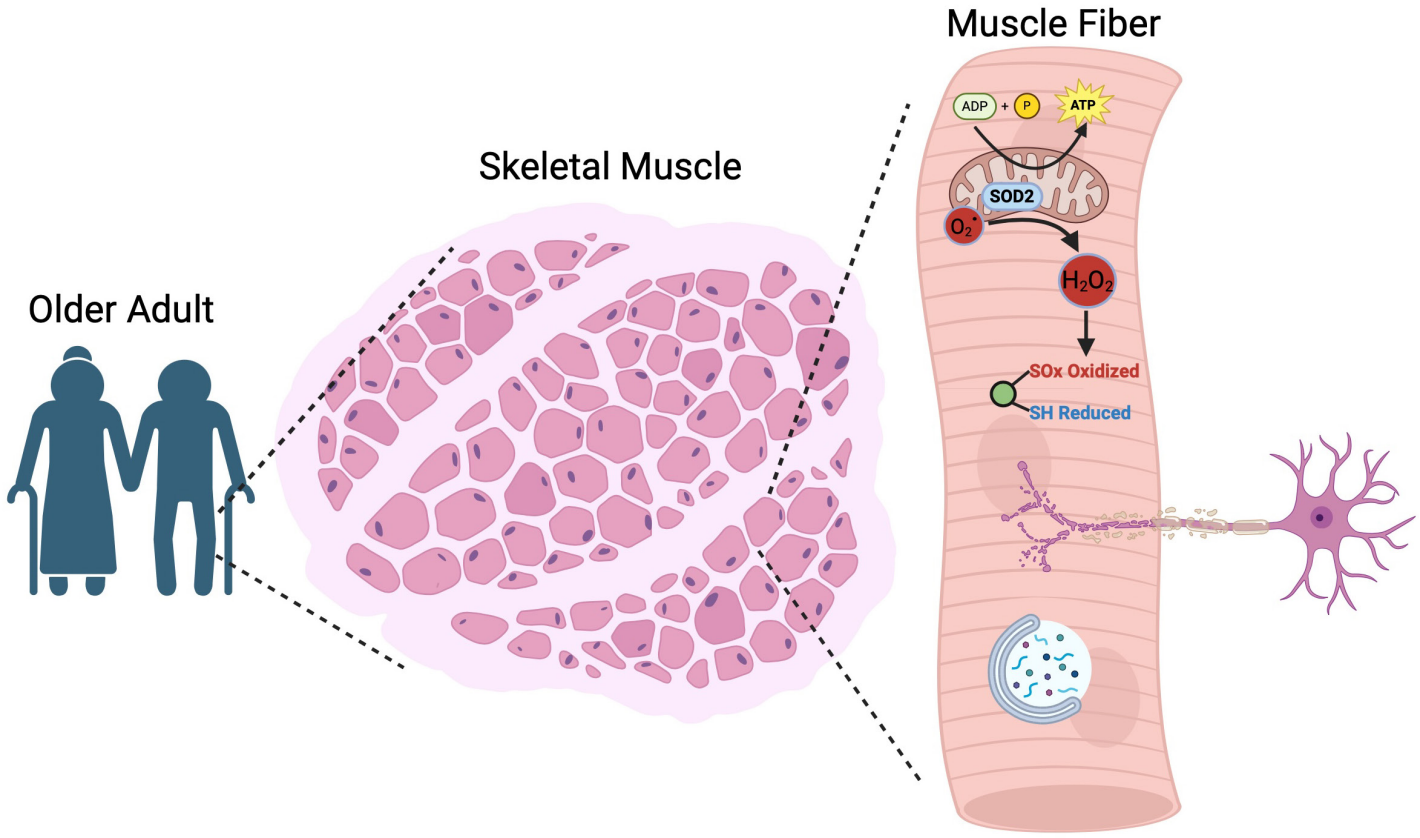
Using assays of samples of skeletal muscle from the vastus lateralis, studies from SOMMA show that mitochondrial generation of ATP, oxidative damage, reduced antioxidant defences (e.g. SOD2), autophagy, and denervation play important roles in mobility folder adults.

Using the gene expression data from muscle in SOMMA, Coen et al. (2024) found that expression of genes involved in autophagy was associated tests of mobility, fitness and strength and also mitochondrial energetics. Autophagy is the cellular process of lysosomal degradation and recycling of damaged cytoplasmic components, including mitochondria (mitophagy) to maintain cellular homeostasis (Picca et al., 2023; Sandri, 2010). It is also noteworthy that excess ROS may damage elements of autophagy and impair the process (Rodney et al., 2016).

Aging in skeletal muscle is associated with recurring cycles of denervation and reinnervation, but during aging the rate of reinnervation can no longer keep pace leading to accumulation of persistently denervated muscle fibers that eventually is manifested clinically as muscle fiber and whole muscle atrophy. Using the muscle gene expression data, Lukasiewicz et al. (2024) showed that transcripts that increase with denervation were associated with poorer mobility along with reduced muscle volume.

In aggregate, findings from SOMMA sketch a picture of the intracellular processes in skeletal muscle that lead to loss of mobility with aging (Figure 1). It needs to add other processes and their interactions. SOMMA plans to extend its assays of cellular properties to include, for example, inherited and somatic mitochondrial DNA mutations and cellular senescence. This approach to studying associations between fundamental cellular processes and phenotypes of aging posits that the cellular biology of muscle may also be a window on aging in other cells, tissues and systems.

SOMMA intends to be a resource for the scientific community engaged in research on human aging. It has an wide array of data including assessments from questionnaires, tests of physical and cognitive performance, whole body MR and CT images, and blood‐based biomarkers in a large population of older adults. (Cummings et al., 2023) It has been supplemented by making the same assessments in smaller cohort of individuals aged 30–69 years old. SOMMA is well‐suited for studies of how aging tissues interact and contribute to frailty and multimorbidity. The data collected in SOMMA are publicly available at https://sommaonline.ucsf.edu. The site is updated biannually as new data become available. The amounts of tissue and blood are limited. Proposals for ancillary studies to use these samples require more stringent review and consideration of the impact on the amount of specimen in the repository. SOMMA welcomes collaborations with scientists—particularly young investigators—who are interested in research about human aging.

## AUTHOR CONTRIBUTIONS

Dr. Steven R. Cummings conceived of the commentary, drafted the first and wrote the final submitted drafts. Dr. Paul M. Coen wrote text that was included in the first draft and reviewed and approved the final version. He also developed the figure. Dr. Luigi Ferrucci wrote text about the contributions of other studies beside SOMMA, edited a draft of the paper and approved the final version.

## CONFLICT OF INTEREST STATEMENT

The authors report no conflicts of interest.

## Data Availability

The data collected in SOMMA are publicly available at https://sommaonline.ucsf.edu.
